# The development of the Western Australian Haemodialysis Vascular Access Complexity instrument

**DOI:** 10.1111/jorc.12390

**Published:** 2021-06-28

**Authors:** Linda L. Coventry, Jon Hosking, Evelyn Coral, Mark Jenkins, Chandra P. Salgado Kent, Doris Chan, Wai Lim, Diane E. Twigg, Claire M. Rickard

**Affiliations:** ^1^ School of Nursing and Midwifery Edith Cowan University Joondalup Western Australia Australia; ^2^ Centre for Research in Aged Care Edith Cowan University Joondalup Western Australia Australia; ^3^ Centre for Nursing Research Sir Charles Gairdner Hospital Nedlands Western Australia Australia; ^4^ Alliance for Vascular Access Teaching and Research Menzies Health Institute Queensland Southport Queensland Australia; ^5^ Renal Services, Waitemata District Health Board, North Shore Hospital Auckland New Zealand; ^6^ Haemodialysis Unit, Sir Charles Gairdner Hospital Nedlands Western Australia Australia; ^7^ Centre for Marine Ecosystems Research Edith Cowan University Joondalup Western Australia Australia; ^8^ Department of Renal Medicine Sir Charles Gairdner Hospital Nedlands Western Australia Australia; ^9^ School of Nursing and Midwifery Griffith University Nathan Queensland Australia

**Keywords:** cannulation‐related complications, haemodialysis, instrument development, kidney failure, reliability and validity, vascular access

## Abstract

**Background:**

The Western Australian Haemodialysis Vascular Access Classification instrument was developed to classify the cannulation complexity of the arteriovenous fistula or arteriovenous graft as simple, challenging, or complex. Although the instrument was developed by experts in haemodialysis nursing, the instrument had not undergone formal validity or reliability testing.

**Objectives:**

Evaluate the Western Australian Haemodialysis Vascular Access Classification instrument for content validity, interrater and test–retest reliability.

**Design:**

Prospective cohort study.

**Participants:**

Content validity was assessed by haemodialysis nursing experts (*n* = 8). The reliability testing occurred in one in‐centre and one satellite haemodialysis unit in Western Australia from September to November 2019. Reliability testing was performed by 38 haemodialysis nurses in 67 patients receiving haemodialysis and 247 episodes of cannulation.

**Measurements:**

Interrater and test–retest reliability assessment was conducted using *κ*, adjusted *κ*, Bland–Altman plots, intraclass correlation coefficient and Pearson's correlation coefficient.

**Results:**

The final version of the instrument (*n* = 20 items) had individual item‐level content validity indices ranging from 0.625 to 1.00 with a scale‐level content validity index of 0.89. For both interrater (*n* = 172 pairs) and test–retest (*n* = 101 pairs), most individual variables had excellent adjusted *κ* (*n* = 33 variables), some fair to good agreement (*n* = 6 variables) and one variable with poor agreement. The classification of simple, challenging and complex demonstrated adjusted *κ* of fair to good, to excellent agreement for interrater reliability with lower levels of agreement for test–retest reliability.

**Conclusions:**

This instrument may be used to match a competency‐assessed nurse to perform the cannulation thereby minimising the risk of missed cannulation and trauma.

## INTRODUCTION

Kidney failure is defined as the permanent loss of kidney function wherein kidney replacement therapy (KRT) is required to sustain life. An estimated 2.6 million people receive KRT worldwide and this is projected to double to 5.4 million by 2030 (Liyanage et al., 2015). Use of KRT through peritoneal dialysis, kidney transplantation or haemodialysis (HD) is a costly but lifesaving treatment. In Australia, HD is the treatment modality of choice for 78% (*n* = 9557 patients) of prevalent dialysis patients (ANZDATA Registry, 2019). Patients on maintenance HD require well‐functioning vascular access (VA) to achieve effective therapy. Maintaining the patency of the VA is an important patient‐centred outcome established by the international Standardized Outcomes in Nephrology initiative (Viecelli et al., 2018). Repeated missed cannulation may result in serious complications such as haematoma, infection, and aneurysm formation, which can lead to need for access revision, central venous line placement, or loss of access (Al‐Jaishi et al., 2017; Harwood et al., 2017; Lee et al., 2006; McCann et al., 2009; Polkinghorne et al., 2013; Schinstock et al., 2011; Vachharajani, 2014; Van Loon et al., 2009; Wilson et al., 2010). Additionally, further cannulation attempts are painful for the patient (Wilson & Harwood, 2017). It is therefore important to develop a HD instrument that can measure VA cannulation complexity, so that cannulation of the VA can successfully occur on the first attempt, resulting in less risk of complications for the patient.

## LITERATURE REVIEW

The Western Australia Haemodialysis Vascular Access Classification (WAHVAC) instrument was developed by a subgroup of the Western Australian (WA) Unit Leaders' Group, comprising of seven HD nurse experts representing the 22 rural, remote and metropolitan WA dialysis centres. The instrument was introduced as part of routine clinical care in all WA HD units from 2011 (J. Hosking, personal communication, September 2019). This instrument aims to classify the cannulation complexity of the arteriovenous fistula (AVF) or arteriovenous graft (AVG) as simple, challenging, or complex. Depending on the classification of complexity, a suitably skilled, competency‐assessed nurse can be matched to perform the cannulation to minimise the risk of missed cannulation and trauma. At our HD centres, we have a rigorous program of competency assessment for access cannulation. This learning framework involves the staff member using Self‐Directed Learning Packages (SLDP) together with Formative and Summative Assessments supervised by expert team members to demonstrate the advancement of their skills and associated knowledge of VA management. Nurses work their way through 3 levels (SDLP's/Assessment) of competency: simple, challenging, complex. Not all nurses will progress to Complex as not all nurses are able to develop a high level of competency.

Our previous study showed that cannulating a fistula compared with a graft, absence of a stent and matured access were associated with successful cannulation (Coventry et al., 2019). The WAHVAC includes these variables in the instrument as well as other variables that impact cannulation complexity.

Instruments that assess access complexity for the related but different scenario of peripheral intravenous catheter insertion are common (Civetta et al., 2019; Hirani et al., 2019; Pagnutti et al., 2016; Van Loon et al., 2019). There is currently no “gold standard” for grading the complexity of HD VA and no published instrument to assess HD VA complexity. Proxy measures for the complexity of HD VA may include the number of cannulation attempts, the need to ask a more experienced nurse to perform the cannulation, inability to complete the prescribed dialysis session, or use of a central venous catheter (CVC) (Coventry et al., 2019). Although the WAHVAC was developed by experts in HD nursing, the instrument had not undergone formal validity or reliability testing. For this study, a panel of VA experts were invited to participate and assess content validity testing of the instrument, and reliability testing was also conducted. Therefore, the aim of this study was to evaluate the WAHVAC instrument for content validity and interrater and test–retest reliability.

## MATERIAL AND METHODS

### Design

A cohort study design was used to assess the reliability of the instrument.

### Participants

#### Content validity

Content validity was conducted by an international panel of experts in HD nursing (*n* = 8). Experts were identified by the authors through publications in the area of HD VA, the Australian New Zealand Vascular Access Nurse network, personal communication and known expertise in cannulation. An email was sent inviting the experts to participate.

#### Reliability

Reliability assessments were conducted using data collected from one in‐centre and one satellite HD unit in Perth, Western Australia from September to November 2019. The participants were HD patients (*n* = 67) with a VA (AVF or AVG) and HD nurses (*n* = 38) who were responsible for cannulating the access. To assess interrater reliability, two nurses completed the WAHVAC instrument independently for the same patient at the same time. To assess test‐retest reliability, the same nurse completed a second WAHVAC instrument for the same patient at a second time point approximately 2 weeks later.

### WAHVAC

**Figure 1:**
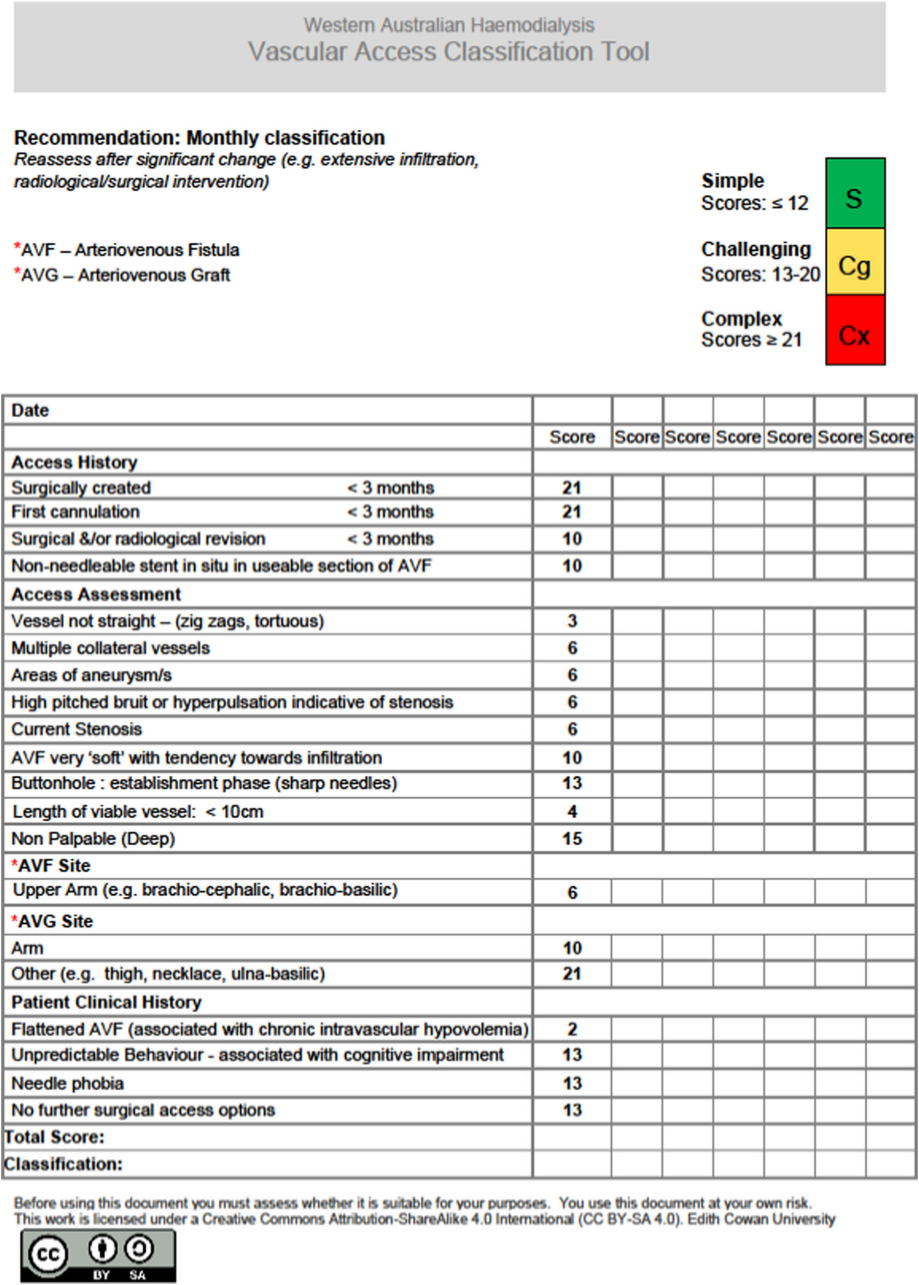
Western Australian Haemodialysis Vascular Access Classification tool

The WAHVAC (see Figure 1) has 20 items that assess access history, access assessment and relevant patient clinical history. The total instrument can be scored from 0 to 209. Each of the variable scores is added together for a total score. Total scores less than or equal to 12 are classified as simple access, scores between 13 and 20 are classified as challenging, and, scores ≥ 21 are classified as complex. The value of the score is a set value, for example, if the access was surgically created less than 3 months ago, the score is 21 and the access is then classified as a complex cannulation. We recommend the complexity of the VA be assessed monthly and reassessed after a significant change, for example, after widespread infiltration or radiological or surgical intervention.

### Data collection

#### Content validity

An international panel of experts conducted content validity assessment of the WAHVAC. Descriptive characteristics of the content validity experts (demographic data; work history, education, and HD training experience) along with the content validity scores were assessed using an electronic survey via email. In addition, the expert panel were asked to comment on the (a) clarity and wording of items, (b) comprehensiveness of the items in reflecting VA complexity, (c) any items to omit, (d) areas for possible improvements or modifications, and, (e) if they agreed with the complexity score allocated to each variable on the WAHVAC. The WAHVAC was sent to the experts on two separate occasions to review the content validity of the items on the instrument.

#### Reliability

A research nurse collected patient and nurse demographic data at study entry.

##### Patient characteristics

The patient data included demographics, medications, and access history collected via a written survey.

##### Nurse characteristics

The nurse data included demographics, work history, education, and HD training experience, collected via a written survey.

##### Episodes of cannulation

The WAHVAC was completed by the HD nurse who used nurse clinical judgement to identify if the variables were present or not present on the WAHVAC. Data on episodes of cannulation included: nurse confidence (before cannulation) with successful cannulation on a scale of 0 (*no confidence*) to 10 (*complete confidence*); if swelling, bruising or haematoma were present at the VA site; if a tourniquet or ultrasound was used; type of cannulation (area, rope ladder or both), the distance between arterial and venous needles; and if an arterial needle was inserted antegrade. Other outcome measures included if an existing CVC was used; if the allocated nurse did cannulate; if another nurse assisted with the cannulation; if dialysis was disrupted or unable to be completed due to cannulation issues; final online clearance (Kt/V); and the number of cannulation attempts. Cannulation episode success was defined as insertion of two needles (arterial and venous) without extra attempts.

The research nurse coordinated the HD nurses to enable two nurses to independently complete the WAHVAC for interrater reliability assessment. The research nurse also coordinated the same HD nurse to complete the WAHVAC for test–retest reliability assessment after a 2‐week period.

#### Ethical considerations

Human research ethics approval was obtained from the study sites and the project team's university (Sir Charles Gairdner Osborne Park Health Care Group, HREC No: 2015‐049; Joondalup Health Campus, HREC No: 1513; and Edith Cowan University, HREC No: 13153). The research nurse explained the study, provided an information sheet and obtained written consent from both patients and nurses to participate in the study.

#### Statistical analyses

Summary statistics, including means and standard deviations (*SD*) or medians and interquartile ranges, were provided for all continuous variables, and frequencies and percentages for all categorical variables. All descriptive statistical analyses were undertaken using SPSS version 27 (IBM Corp. Released 2015, IBM SPSS Statistics for Windows, Version 26.0; IBM Corp). *κ* analyses were performed using Microsoft Excel (version 1908; Microsoft). The Consensus‐based Standards for the Selection of Health Measurement Instruments (COSMIN) study design checklist recommends for studies assessing *κ*, sensitivity and specificity, the sample size should be >100 (Mokkink et al., 2019). For reliability assessment, 100 pairs are recommended (Mokkink et al., 2019).

##### Content validity

Expert reviewer characteristics were analysed using descriptive characteristics. An a priori acceptable level of interrater agreement for relevancy was set at 0.70 and higher (Mojahedi et al., 2014). The content validity index (CVI) was computed to derive the CVI for each item (I‐CVI) in the scale. The I‐CVI was calculated as the proportion of experts rating either a 3 or 4 (*not relevant* = 1, *somewhat relevant* = 2, *quite relevant* = 3, and *very relevant* = 4), divided by the total number of experts who rated the item. The I‐CVIs between 0.7 and 1.0 inclusive were retained, the I‐CVIs between 0.5 and 0.7 were further revised or clarified, and the I‐CVIs <0.5 were discarded. Derivation of the overall instrument was expressed as the number of items rated three or four by at least 80% of the experts. Scale‐level content validity (S‐CVI), the proportion of items given a rating of three or four by all the raters, was derived from averaging across all I‐CVIs. Also reported was the number of variables where there was total expert agreement and S‐CVI universal agreement (S‐CVI UA), and this was calculated by dividing the number of variables with total expert agreement by the number of variables in the instrument.

##### Reliability

To assess the interrater and test–retest reliability of each of the individual variables (present, not present) and the classifications of simple, challenging and complex on the WAHVAC we conducted observed and expected agreements, *κ* statistics and their 95% confidence intervals (CI), the bias index, prevalence index and the adjusted *κ* value. See Supporting Information 1 for formulae calculations. Multiple studies (Byrt et al., 1993; Viera & Garrett, 2005) have reported that the *κ* statistic is not always satisfactory for assessing agreement, and recommend that bias and prevalence be taken into account when the magnitude of one or both of their indices is close to one. Bias, prevalence indices, and the prevalence‐adjusted bias‐adjusted *κ* (referred to as adjusted *κ*) were calculated (Byrt et al., 1993). For interpretation purposes, a *κ* (and adjusted *κ*) scale was used (poor, <0.40; fair to good, 0.40–0.75; excellent, >0.75) (Fleiss, 1981).

The reliability of the total score of the WAHVAC (range: 0–209) was assessed using intraclass correlation coefficients (ICCs), Bland–Altman plots, and linear regression was used to investigate evidence of proportional bias. To calculate ICC, a two‐way random effect model was calculated. For the regression analysis to assess proportional bias, the variables assessed were the difference in score total between the two nurses, and the mean total score for the two nurses. A scatter plot was used to investigate the relationship between the total scores of the interrater reliability as well as the test–retest reliability, and Pearson's product moment correlation coefficient was reported. Preliminary analyses were performed to ensure no violation of the assumptions of normality, linearity and homoscedasticity.

For interpretation purposes, an ICC scale was used (poor, <0; slight, 0.01–0.20; fair, 0.21–0.40; moderate, 0.41–0.60; substantial, 0.61–0.80; almost perfect, 0.81–1.00) (Landis & Koch, 1977). For interpretation purposes, a Pearson *r* scale was used (poor, 0.00–0.29; fair, 0.30–0.59; moderately strong, 0.60–0.79; very strong, 0.80–1.00) (Chan, 2003).

## RESULTS

### Content validity WAHVAC

Demographic data of the HD nurse experts (*n* = 8) are presented in Table 1. On the first occasion, the WAHVAC instrument was sent to the experts, the original 35‐item instrument was reduced to a 24‐item instrument. On the second expert review, the instrument was further reduced to 20 items. The wording of the items was clarified and scores for individual variables were adapted on the advice of the experts. The final instrument reports I‐CVI > 0.75 for 19 items with one item at 0.62. The S‐CVI was 0.89 and S‐CVI UA 0.40 (see Supporting information Appendix 1 and Supporting Information Material 2).

**Table 1:** Demographic characteristics of content validation experts (*N* = 8)

	Mean (*SD*)	Range
Age (years)	51.7 (7.9)	42–63
Years as registered nurse	31.8 (7.0)	23–40
Years as haemodialysis nurse	23.9 (7.2)	15–35

	*n*	%
Employment status
Full time	7	87.5
Part time	1	12.5
Sex		
Female	7	87.5
Male	1	12.5
Highest level of education
Postgraduate	3	37.5
Master	4	50.0
PhD	1	12.5
Job title		
Clinical nurse consultant	2	25.0
Nurse unit manager	2	25.0
Director	1	12.5
Academic	3	37.5
Country of residence
Australia
Western Australia	2	25.0
Queensland	2	25.0
New South Wales	1	12.5
Victoria	1	12.5
United States of America	2	25.0

### Reliability of the WAHVAC

#### Patient and nurse characteristics

The demographic data of the patient and nurse characteristics are presented in Table 2.

**Table 2:** Patient (n=67) and nurse (n=38) characteristics for Reliability assessment

Patient variables	Mean (SD)	Range	
Age (years)	67.5 (13.7)	25.9 – 92.9	
BMI (kg/m^2^)	26.6 (5.6)	17.9 – 39.8	
Age of access (years)	4.6 (5.3)	0.1 – 32.7	
	Median	IQR	
	3.0	1.7 – 5.3	
		n	(%)
Sex			
Female		21	(31.3)
Male		46	(68.7)
Medications			
Steroids		5	(7.5)
Immunosuppressant		2	(3.0)
Anticoagulantn		20	(29.9)
PAI		24	(36.4)
Nurse characteristics	Mean (SD)	Range	
Age (years)	47.4 (8.7)	24 – 61	
Years as registered nurse	18.8 (9.7)	1 – 40	
Years as haemodialysis nurse	14.7 (7.4)	1 – 30	
		n	(%)
Sex			
Female		33	(86.8)
Male		5	(13.2)
Employment status			
Full time		13	(34.2)
Part time/casual		25	(65.8)
Highest level of education			
RN hospital certificate		3	(7.9)
RN diploma		6	(15.8)
BScN/BN		11	(28.9)
RN post‐basic certificate		1	(2.6)
Graduate certificate		9	(23.7)
Graduate diploma		5	(13.2)
Master's degree		3	(7.9)
Job title			
RN		28	(73.7)
CN/SDN		10	(26.3)
Post‐graduate in renal nursing			
Yes		21	(55.3)
No		17	(44.7)

Abbreviations: BMI, body mass index; BN, Bachelor of Nursing; BScN, Bachelor of Science in Nursing; CN, clinical nurse; IQR, interquartile range; PAI, platelet aggregation inhibitor; RN, registered nurse; SDN, staff development nurse.

#### Episodes of cannulation

During the 12‐week study period, there were 247 episodes of VA cannulation in 67 HD patients, performed by 38 dialysis nurses. The average number of episodes of cannulation per patient was 3.7 (*SD* = 2.3). Successful cannulation at first attempt occurred in most cannulation episodes (*n* = 236, 95.5%) with the miscannulation rate small (*n* = 11, 4.4%). The 11 miscannulations occurred in nine patients; therefore 13.4% of patients had a least one event of miscannulation. The complexity categories were mostly evenly distributed among simple (*n* = 87, 35.2%), challenging (*n* = 68, 27.5%) and complex (*n* = 92, 37.2%) categories. Other characteristics of cannulation are presented in Table 3.

**Table 3:** Characteristics of cannulation (*n* = 247) and variables in the WAHVAC instrument

	*n*	Mean (*SD*)	Range
**Characteristics of cannulation**			
Confident successful first attempt	242	9.2 (1.3)	5–10
Distance between arterial and venous site (cm)	234	6.3 (3.4)	2–40
Final Online clearance Kt V	246	1.33 (0.17)	0.78–1.85

	*N*	*n*	%
Complexity categories			
Simple	247	87	35.2
Challenging	247	68	27.5
Complex	247	92	37.2
Swelling present at cannulation sites	242	5	2.1
Bruising present at cannulation sites	240	26	10.8
Hematoma present at cannulation sites	242	5	2.1
Tourniquet used	245	211	86.1
Ultrasound used	243	13	5.3
Cannulation			
Area	231	37	16.0
Rope ladder		119	51.5
Both		75	32.5
Arterial needle antegrade insertion	244	186	76.2
Did the allocated nurse cannulate?	247	231	93.5
Did another nurse assist with cannulation?	247	13	5.3
Number of cannulation attempts (artery)			
1	247	239	96.8
2		7	2.8
3		1	0.4
Number of cannulation attempts (vein)
1	247	240	97.2
2		6	2.4
3		1	0.4
An existing CVC used	247	1	0.4
Dialysis disrupted due to cannulation issues	247	5	2.0
Unable to dialysis patient due to cannulation issues	247	0	0.0
**Characteristics of WAHVAC instrument**			
Surgically created <3 months	247	4	1.6
First cannulated <3 months	247	18	7.3
Surgical revision <3 months	247	38	15.4
Nonneedleable stent in situ in useable section of AVF	247	3	1.2
Vessel not straight (zig zags, tortuous)	247	68	27.5
Multiple collateral vessels	247	19	7.7
Areas of aneurysm/s	247	148	59.9
Has high pitched bruit or hyperpulsation indicative of stenosis	247	29	11.7
Current stenosis	247	14	5.7
AVF very “soft” with tendency towards infiltration	247	36	14.6
Buttonhole: establishment phase (sharp needles)	247	0	0.0
Length of viable vessel: <10 cm	247	130	52.6
Nonpalpable (deep)	247	4	1.6
Upper Arm (e.g. brachio‐cephalic, brachio‐basilic)	247	151	61.1
Arm	247	11	4.5
Other (e.g., thigh, necklace, ulna‐basilic)	247	0	0.0
Flattened AVF (associated with chronic intravascular hypovolemia)	247	1	0.4
Unpredictable behaviour—associated with cognitive impairment	247	11	4.5
Needle phobia	247	19	7.7
No further surgical access options	247	7	2.8

Abbreviations: AVF, arteriovenous fistula; CVC, central venous catheter; WAHVAC, Western Australia Haemodialysis Vascular Access Classification.

#### Reliability assessment

##### Interrater reliability

The interrater reliability conducted using 172 pairs of observations are presented in Table 4. Although bias had little effect in this study, the prevalence index was high for most symptoms and justified the use of the adjusted *κ*. Most variables (*n* = 17, 85.0%) had adjusted *κ* statistics >0.75, indicating excellent agreement. The adjusted *κ* values for the classifications ranged from excellent, to fair to good (simple, 0.79; challenging, 0.65; complex, 0.70). For the WAVHAC total score, the ICC was 0.76, *F*(45, 45) = 4.21, *p* < 0.001, 95% CI: 0.57–0.87, indicating substantial interrater agreement at time 1 between nurse 1 and nurse 2 (results not reported in tables). There were 9 of 172 occasions where a result was outside of the 95% confidence intervals (see Supporting Information Material 3). No statistical significance was found using linear regression (*β* coefficient = −0.09; a value close to 0; *p* = 0.26), indicating no proportional bias between the difference in score total between the two nurses, and the mean total score for the two nurses. There was a very strong, positive correlation of the WAHVAC total score from Nurse 1 and Nurse 2 (*r* = 0.81; *n* = 172; *p* < 0.001) (see Supporting Information Material 5).

**Table 4:** Interrater reliability of the WAHVAC instrument (*n* = 172)

Variable	Observed agreement, %	Expected agreement, %	*κ* (95% CI)	Bias index^a^	Prevalence index^b^	Adjusted *κ*
Access history
Surgically created <3 months	100.0	96.6	1.00 (0.85–1.15)	0.00	0.97	1.00
First cannulated <3 months	98.8	83.1	0.93 (0.78–1.08)	0.01	0.81	0.98
Surgical revision <3 months	97.7	72.8	0.91 (0.77–1.06)	0.00	0.67	0.95
Non‐needleable stent in situ in useable section of AVF	97.7	96.6	0.32 (0.17–0.47)	0.00	0.97	0.95
Access assessment						
Vessel not straight (zig zags, tortuous)	82.0	56.2	0.59 (0.44–0.74)	−0.04	0.35	0.64
Multiple collateral vessels	93.6	83.5	0.61 (0.47–0.76)	0.04	0.82	0.87
Areas of aneurysm/s	85.5	52.3	0.70 (0.55–0.84)	0.02	−0.22	0.71
Has high pitched bruit or hyperpulsation indicative of stenosis	91.3	78.1	0.60 (0.45–0.75)	0.01	0.75	0.83
Current stenosis	93.0	88.0	0.42 (0.27–0.57)	0.01	0.87	0.86
AVF very “soft” with tendency towards infiltration	89.5	71.2	0.64 (0.49–0.79)	0.00	0.65	0.79
Buttonhole: establishment phase (sharp needles)	100.0	100.0	–	0.00	1.00	1.00
Length of viable vessel: <10 cm	83.7	50.0	0.67 (0.56–0.78)	−0.06	−0.06	0.67
Nonpalpable (deep)	96.5	95.4	0.23 (−0.17 to 0.64)	0.02	0.95	0.93
AVF site
Upper Arm (e.g., brachio‐cephalic, brachio‐basilic)	94.8	51.4	0.89 (0.82–0.96)	−0.02	−0.17	0.90
AVG site						
Arm	99.4	89.6	0.94 (0.84–1.05)	0.01	0.89	0.99
Other (e.g. thigh, necklace, ulna‐basilic)	100.0	100.0	–	0.00	1.00	1.00
Patient clinical history
Flattened AVF (associated with chronic intravascular hypovolemia)	98.8	98.8	0.00 (−0.01 to 0.01)	−0.01	0.99	0.98
Unpredictable behaviour—associated with cognitive impairment	97.1	89.6	0.72 (0.49–0.95)	−0.01	0.89	0.94
Needle phobia	96.5	89.0	0.68 (0.44–0.92)	0.00	0.88	0.93
No further surgical access options	96.5	94.3	0.38 (−0.01 to 0.78)	0.01	0.94	0.93
Classification						
Simple	89.5	58.3	0.75 (0.60–0.90)	0.00	0.41	0.79
Challenging	82.6	57.8	0.59 (0.44–0.74)	−0.03	0.40	0.65
Complex	84.9	51.9	0.69 (0.54–0.83)	0.04	0.20	0.70

Abbreviations: AVF, arteriovenous fistula; AVG, arteriovenous graft; BI, bias index; CI, confidence interval; PI, prevalence index; WAHVAC, Western Australian Haemodialysis Vascular Access Complexity.

^a^ The BI is the difference in the proportions of “yes” between the two nurse assessors; it has a minimum of −1 and maximum of 1.

^b^ The PI is the difference in prevalence of “yes” and “no”, prevalence being calculated as means for the two nurse assessors. PI has a minimum of ‐1 and a maximum of 1 and is 0 when the mean prevalence of “yes” is 50%.

##### Test–retest reliability

The test‐retest reliability conducted using 101 pairs of observations are presented in Table 5. The mean time between the test and the retest reliability was 19.6 (*SD* = 13.5) days. Most variables (*n* = 16, 80.0%) had adjusted *κ* statistics >0.75, indicating excellent agreement. The adjusted *κ* values for the classifications ranged from poor, to fair to good (simple, 0.52; challenging, 0.23; complex, 0.62). For the WAVHAC total score, the ICC was 0.78, *F*(53, 53) = 4.66, *p* < 0.001, 95% CI: 0.63–0.88) indicating substantial test‐retest agreement between the same nurse at time 1 and time 2 (results not reported in tables). There were 8 of 101 occasions where a result was outside of the 95% CIs (see Supporting Information Material 4). No statistical significance was found using linear regression (*β* coefficient = 0.06; a value close to zero; *p* = 0.52), indicating no proportional bias between the difference in score total between the two nurses, and the mean total score for the two nurses. There was a moderately strong, positive correlation of the WAHVAC total scores from the same nurse at Time 1 and Time 2 (*r* = 0.68; *n* = 101; *p* < 0.001 [see Supporting Information Material 6]).

**Table 5:** Test–retest reliability of the WAHVAC instrument (*n* = 101)

Variable	Observed agreement, %	Expected agreement, %	*κ* (95% CI)	Bias index^1^	Prevalence index^2^	Adjusted *κ*
Access history
Surgically created <3 months	99.0	97.1	0.66 (0.04–1.29)	−0.01	0.97	0.98
First cannulated <3 months	95.1	91.4	0.43 (0.01–0.85)	−0.05	0.91	0.90
Surgical revision <3 months	91.1	71.2	0.69 (−1.22 to 2.59)	−0.05	0.65	0.82
Nonneedleable stent in situ in useable section of AVF	99.0	99.0	0.00 (−0.01 to 0.01)	0.01	0.99	0.98
Access assessment						
Vessel not straight (zig zags, tortuous)	83.2	57.0	0.61 (−1.08 to 2.29)	−0.05	0.38	0.66
Multiple collateral vessels	91.1	86.2	0.35 (0.02–0.68)	0.03	0.85	0.82
Areas of aneurysm/s	87.1	55.0	0.71 (0.57–0.86)	‐0.03	‐0.32	0.74
Has high pitched bruit or hyperpulsation indicative of stenosis	87.1	83.0	0.24 (−0.05 to 0.54)	−0.01	0.81	0.74
Current stenosis	94.1	90.6	0.37 (−0.03 to 0.77)	0.00	0.90	0.88
AVF very “soft” with tendency towards infiltration	89.1	81.4	0.42 (0.13–0.70)	−0.01	0.79	0.78
Buttonhole: establishment phase (sharp needles)	100.0	100.0	–	0.00	1.00	1.00
Length of viable vessel: <10 cm	65.4	50.5	0.28 (0.09–0.47)	−0.15	‐0.18	0.31
Nonpalpable (deep)	99.0	95.2	0.80 (0.40–1.19)	−0.01	0.95	0.98
AVF site
Upper arm (e.g., brachio‐cephalic, brachio‐basilic)	92.1	52.9	0.83 (0.72–0.94)	0.06	‐0.25	0.84
AVG site						
Arm	99.0	95.2	0.80 (0.40–1.19)	−0.01	0.95	0.98
Other (e.g., thigh, necklace, ulna‐basilic)	100.0	100.0	–	0.00	1.00	1.00
Patient clinical history
Flattened AVF (associated with chronic intravascular hypovolemia)	99.0	99.0	0.00 (−0.01 to 0.01)	0.01	0.99	0.98
Unpredictable behaviour—associated with cognitive impairment	97.0	93.3	0.56 (0.10–1.01)	−0.01	0.93	0.94
Needle phobia	98.0	88.8	0.82 (0.58–1.06)	0.02	0.88	0.96
No further surgical access options	96.0	92.3	0.48 (0.04–0.92)	0.02	0.92	0.92
Classification						
Simple	76.2	55.5	0.47 (0.26–0.65)	0.10	0.35	0.53
Challenging	61.4	53.7	0.17 (−0.03 to 0.36)	−0.05	0.28	0.23
Complex	81.2	57.0	0.56 (0.37–0.76)	−0.05	0.38	0.62

Abbreviations: AVF, arteriovenous fistula; AVG, arteriovenous graft; BI, bias index; CI, confidence interval; PI, prevalence index; WAHVAC, Western Australian Haemodialysis Vascular Access Complexity.

^1^ The BI is the difference in the proportions of “yes” between the two nurse assessors; it has a minimum of −1 and a maximum of 1.

^2^ The PI is the difference in prevalence of “yes” and “no,” prevalence being calculated as means for the two nurse assessors. PI has a minimum of −1 and a maximum of 1 and is 0 when the mean prevalence of “yes” is 50%.

## DISCUSSION

This is the first study to develop a valid and reliable HD VA complexity instrument. Maintenance of VA remains the Achilles' heel of HD treatment. Repeated missed cannulation results in serious complications and increases the risk of permanent loss of dialysis access (Harwood et al., 2017; Schinstock et al., 2011; Vachharajani, 2014; Van Loon et al., 2009). The development of a VA cannulation complexity instrument has the potential to minimise the risk of missed cannulation and trauma by matching each patient to a suitably skilled, competency‐assessed nurse to perform the cannulation. Our study confirms the content validity of the WAHVAC to grade the complexity of an AVF or AVG for cannulation (S‐CVI = 0.89). The study also reports fair to good, to excellent interrater reliability for the classification of groups into simple, challenging, complex. The majority of individual WAHVAC variables (*n* = 17, 85.0%; and *n* = 16, 80.0%, respectively) also had excellent agreement for interrater and test–retest reliability (adjusted *κ* statistics >0.75). The WAHVAC has an advantage over other instruments that assess peripheral intravenous cannulation complexity for the insertion of catheters (Civetta et al., 2019; Hirani et al., 2019; Pagnutti et al., 2016; Van Loon et al., 2019) as the variables on the instrument are specific to HD VA.

According to Polit and Beck (2006), for a scale to have excellent content validity, the S‐CVI should be 0.90 or higher. The WAHVAC reported an S‐CVI of 0.89 due to the inclusion of the variable “needle phobia” (I‐CVI of 0.62). The authors considered it important to include this variable because in cases of true needle phobia, patients may experience vaso‐vagal episodes, including symptoms such as hypotension, nausea and dizziness (Mott & Moore, 2009). Additionally, the nurse may need to manage potential issues such as the patient withdrawing the AVF arm without warning, vocalising loudly and feeling faint. Also, a hyper‐anxious patient can directly impact the success of a less assured nurse conducting the cannulation. Conclusions from a Kidney Disease Improving Global Outcomes conference also highlighted needle phobia as a potential barrier to home‐based or self‐care dialysis (Chan et al., 2019).

Over 80% of the individual WAHVAC variables had excellent agreement for both interrater and test–retest reliability (*n* = 17, 85.0%; and n = 16, 80.0%, respectively). However, for both interrater and test–retest reliability, three variables reported adjusted *κ* values as fair to good agreement. These variables were vessel not straight (zig‐zags), areas of aneurysm and length of the viable vessel <10 cm. Of note, for test–retest for the variable length of the viable vessel, <10 cm scored a poor adjusted *κ* of 0.31. The most likely reason for this may suggest the length of the viable vessel may change over time. Even though tape measures were available, a more accurate assessment of “usable” vessel could be attained by using ultrasound, however, ultrasound was rarely used in this study (*n* = 13, 5.3%). The length of the viable vessel may be affected by bruising and infiltration, after infiltration, it is recommended the patient be cannulated downstream of the infiltration thus reducing the length of the viable vessel (Daugirdas et al., 2015). Bruising with hematoma may also compress the vessel and be painful to touch for the patient (Inglese, 2017). The mean time between the first assessment (test 1) and the second assessment (retest) was 19 days. Therefore, it is highly possible for the length of the viable vessel to change over a 2–3‐week period.

The interrater and test–retest reliability for categories of simple, challenging and complex was reported as fair to good, to excellent, based on the adjusted *κ* values. The exception was for the test‐retest reliability for the category of challenging, which had poor adjusted *κ*. Potential reasons why the “challenging” category had poor test–retest adjusted *κ* may include the development of bruising, infiltration and hematoma during cannulation which then changes the complexity grade of the access over a short time period. We also acknowledge that HD nurses have different levels of experience with cannulation; what may be challenging to one nurse may be complex to another.

## IMPLICATIONS FOR CLINICAL PRACTICE

There needs to be greater emphasis on ways to achieve successful VA cannulation to promote the best health outcomes for patients on HD. VA is often referred to as the patient's “lifeline” and patency of the access has been identified as a core patient‐centred outcome (Viecelli et al., 2018). The WAHVAC instrument offers a simple and practical approach to reduce the frequency of complications associated with missed cannulation by matching the cannulation complexity of access to an HD nurse with the appropriate level of experience ideally by the use of a structured competency assessment framework. Reduced access complications lead to better patient outcomes and quality of life and there may be an economic benefit through the reduced need for radiological and surgical interventions, as well as, need for central line insertion. Unsuccessful cannulation is burdensome for both the patient and the nurse.

In addition, the WAHVAC gives HD centres and nurses a framework and scope by which to develop education programs and competency assessment in dialysis access cannulation. At our centre, the HD nurses are assessed and deemed competent to cannulate a “simple”, “challenging” or “complex” VA. Along with competency assessment, there is ongoing nurse education and training to ensure best practice based on the latest research data and incorporating new techniques such as the introduction of ultrasound‐guided cannulation as technology advances.

### Implications for research

Further research is required to investigate the reliability of the variables that have low prevalence. To enhance the generalisability, external validity and applicability, the reliability of the instrument should be assessed in other HD units.

### Strengths and limitations

The strengths of this study include the rigour associated with the content validation process to identify the variables associated with complexity of cannulation. Additionally, the reliability data was collected over two sites with a large sample size of both patients and nurses which increases generalisability. We had 172 pairs of observations to assess interrater reliability and 101 pairs of observations to assess test‐retest reliability. A limitation of our study is the low prevalence of some of the items on the WAHVAC instrument which limited assessment of reliability for these variables. For example, there were no patients in the buttonhole establishment phase which can be explained by it being a technique not common in Australia and predominantly used in Europe and Japan (Vachharajani et al., 2020). Another limitation to the study, is the number of area cannulations used, as area cannulations weaken the fistula wall (Parisotto et al., 2014). It is important to avoid area cannulations whenever possible. There were also no thigh, necklace or brachio‐basilic fistula/graft. Additionally, the patients rarely had variables of surgically created less than 3 months, first cannulated less than 3 months, nonneedleable stent in situ, multiple collaterals, nonpalpable (deep) and no further surgical access. Therefore, we recommend further research is needed to establish reliability of the variables of WAHVAC with a low prevalence.

## CONCLUSION

People with kidney failure require KRT to sustain life. Successful cannulation of vascular access is required to deliver HD treatment. This classification instrument allows patients to be matched to a competency‐assessed nurse to perform the cannulation thereby minimising the risk of missed cannulation and trauma.

This study has demonstrated the WAHVAC to be a valid and reliable instrument to assess the complexity of cannulating HD VA. Reliability agreement for individual variables of the WAHVAC varies from fair to good, to excellent with only one variable considered as having poor agreement. Although most variables were found to be valid and reliable, further studies are needed to establish the validity and reliability of low prevalence variables following the widespread application of the WAHVAC.

## CONFLICT OF INTERESTS

The authors declare that there are no conflicts of interest.

## AUTHOR CONTRIBUTIONS

*Principle project leader, conceived study, participated in design and coordination, analysed data, helped to draft manuscript, read and approved the final manuscript*: Linda L. Coventry. *Conceived study, participated in design and coordination, helped to draft manuscript, read and approved the full manuscript*: Jon Hosking and Evelyn Coral. *Helped to analyse the data, helped to draft manuscript, and read and approved the final manuscript*: Mark Jenkins and Chandra P. Salgado Kent. *Helped to draft manuscript, and read and approved the final manuscript*: Doris Chan, Wai Lim, Diane E. Twigg, and Claire M. Rickard.

## Supporting information

Supporting information.
