# Supplementary material for: The development of the Western Australian Haemodialysis Vascular Access Complexity instrument
Source: J Ren Care. 2021 Jun 28;48(3):185–96. doi: 10.1111/jorc.12390 (PMC9543205; doi:10.1111/jorc.12390)
Supplement: Supplementary file 1 — Supporting information. [file JORC-48-185-s001.docx]

**Supplementary materials**

*Supplementary material 1. Formulae to Calculate Statistics*

| Variable documented on the first WAHVAC | Variable document on the second WAHVAC | | |
| --- | --- | --- | --- |
|  | Yes | No | Total |
| Yes | A | B | **B**1 = A + B |
| No | C | D | **B**2 = C + D |
| Total | **A**1 = A+ C | **A**2 = B + D | N |

Observed agreement = A + D / N; expected agreement = (**A**1 x **B**1 + **A**2 x **B**2) / N^2^

Sensitivity = A/A1; specificity = D/A2; positive predictive value = A/B1; negative predictive value = D/B2

^†^WAHVAC, Western Australian Haemodialysis Vascular Access Complexity

*Supplementary material 2. Expert reviewers (n=8) content validity WAHVAC*^†^

| **Variable Total in** | **agreement** | **I-CVI**^¶^ |
| --- | --- | --- |
| **Access History** | 8 | 1.00 |
| Surgically created <3 months |  |  |
| First cannulated <3 months | 8 | 1.00 |
| Surgical revision <3 months | 7 | 0.88 |
| Non-needleable stent in situ in useable section of AVF^‡^ | 8 | 1.00 |
| **Access Assessment** | 7 | 0.88 |
| Vessel not straight – (zig zags, tortuous) |  |  |
| Multiple collateral vessels | 7 | 0.88 |
| Areas of aneurysm/s | 8 | 1.00 |
| Has high pitched bruit or hyperpulsation indicative of stenosis | 8 | 1.00 |
| Current stenosis | 8 | 1.00 |
| AVF^‡^ very ‘soft’ with tendency toward infiltration | 8 | 1.00 |
| Buttonhole: establishment phase (sharp needles) | 7 | 0.88 |
| Length of viable vessel: <10cm | 7 | 0.88 |
| Non palpable (deep) | 8 | 1.00 |
| **AVF**^‡^ **Site** | 7 | 0.88 |
| Upper Arm (e.g. brachio-cephalic, brachio-basilic) |  |  |
| **AVG**^§^ **Site** | 6 | 0.75 |
| Arm |  |  |
| Other (e.g. thigh, necklace, ulna-basilic) | 7 | 0.88 |
| **Patient Clinical History** |  |  |
| Flattened AVF^‡^ (associated with chronic intravascular hypovolemia) | 6 | 0.75 |
| Unpredictable Behaviour - associated with cognitive impairment | 6 | 0.75 |
| Needle phobia | 5 | 0.62 |
| No further surgical access options | 6 | 0.75 |
| S-CVI^††^ | | 0.89 |
| Total agreement | | 8/20 |
| S-CVI/UA^‡‡^ | | 0.40 |
| ^†^WAHVAC, Western Australian Haemodialysis Vascular Access Complexity; ^‡^AVF, arteriovenous fistula; ^§^AVG, arteriovenous graft; ^¶^I-CVI, item-level content validity index; ^††^S-CVI, scale-level content validity index; ^‡‡^S-CVI / UA, S-CVI universal agreement calculation method | | |

*Supplementary material 3. Bland Altman Plot of Total Scores for Inter-rater Reliability (n=172)*

**
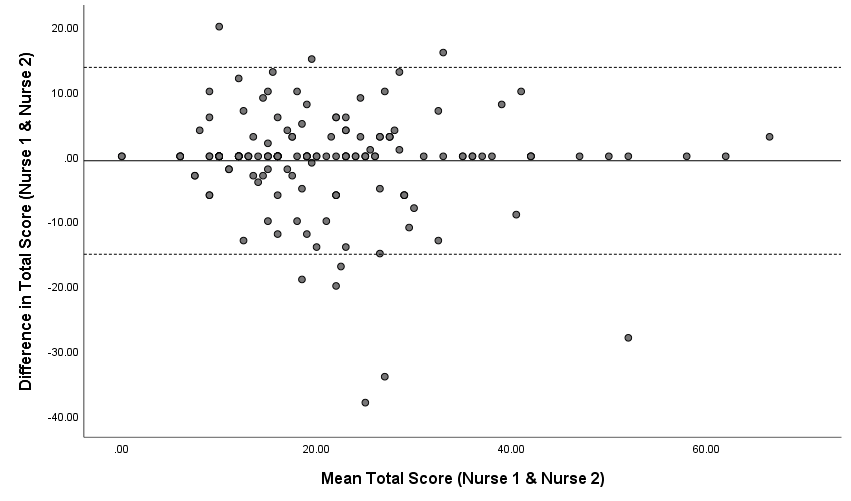
**

*Supplementary material 4. Bland Altman Plot of Total Scores for Test-Retest Reliability (n=101)*

**
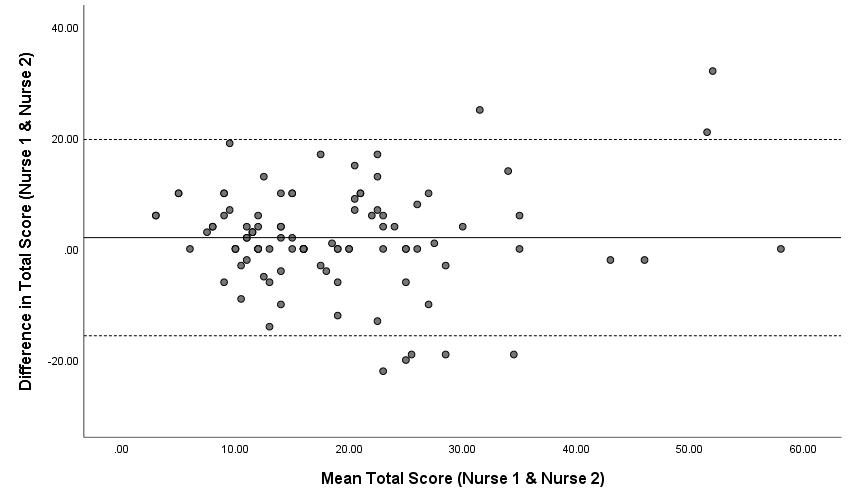
**

*Supplementary material 5. Scatter Plot of Total Scores for Inter-rater Reliability (n=172)*


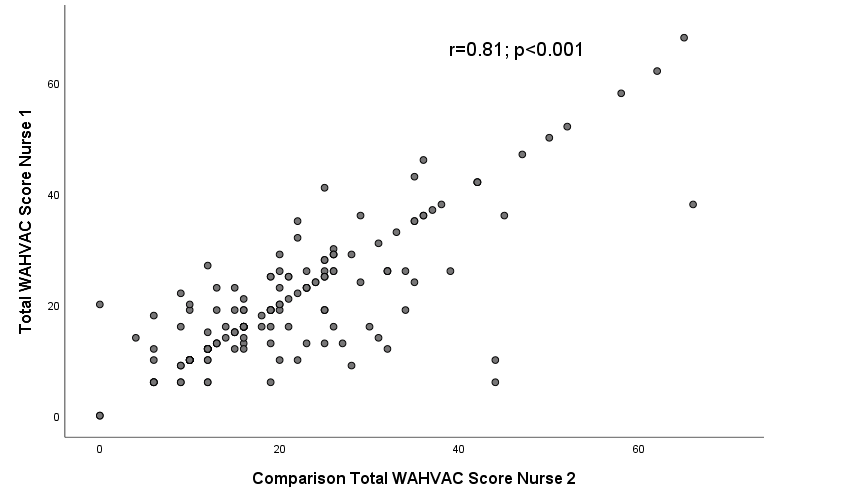


*Supplementary material 6. Scatter Plot of WAHVAC Total Scores for Time 1 & Time 2 (n=101)*

**
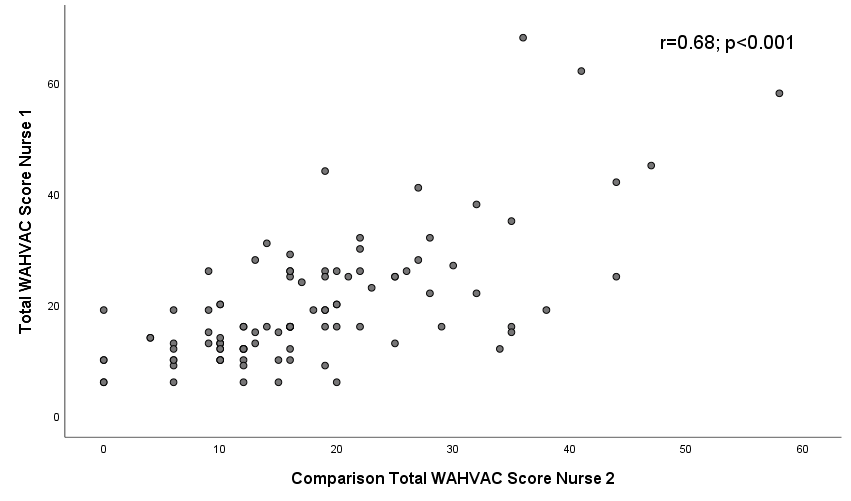
**
